# Mast Cell Leukemia with Ascites and Multiple Organs Damage

**DOI:** 10.30699/ijp.2019.96187.1948

**Published:** 2019-08-01

**Authors:** Elham Jafari, Ali Hadipour, Behjat Kalantari Khandani, Firoozeh Abolhasani

**Affiliations:** 1 *Pathology and Stem Cells Research Center, Dept. of Pathology, Afzalipour Kerman Medical Science University, Kerman, Iran*; 2 *Pathology and Stem Cells Research Center, Dept. of Pathology, Afzalipour Kerman Medical Science University, Kerman, Iran*; 3 *Department of General Surgery, School of Medicine, Afzalipour Kerman University of Medical Sciences, Kerman, Iran*

**Keywords:** Mast cell leukemia, Systemic mastocytosis, Ascites, Multiple organs damage, Prognostic factors

## Abstract

Mast Cell Leukemia (MCL), a rare subtype of systemic mastocytosis is defined by bone marrow involvement as atypical and aleukemic mast cells, if more than 20% and less than 10% of peripheral WBCs are mast cells, respectively.

We met a case of aleukemic MCL presenting with anemia and ascites for 2 years, referred for BM evaluation, suspicious of leukemia. Our findings included BM involvement by diffused aggregates of oval- and spindle-shaped atypical mast cells, lacking mature mast cells and other hematopoietic cells. The mast cells were absent in peripheral blood smear. Further assessments showed positive reaction of mast cells metachromatic granules with Tryptase, Giemsa and Toluidine blue stains, the expression of CD117/KIT and CD45 by immunohistochemistery, and elevated level of serum Tryptase.

Radiologic investigations revealed generalized lymphadenopathy, and massive hepatosplenomegaly, followed by the cervical lymphadenectomy, and liver wedge biopsy. Suspicious peritoneal lesions were identified and underwent excisional biopsy. Microscopic evaluations showed lymph nodes and liver involvement by cancer cells and the same features in peritoneal seeding. Multiple organs damage progressed in few months and the patient died despite surgery and chemotherapy.

In conclusion, we report an extremely rare case of aleukemic MCL with multiple organs damage such as liver, peritoneum, spleen, gastrointestinal tract and BM, presenting by ascites. According to this case and previous parallel studies, we suggest some clinicopathologic features in favor of poor prognosis, including the presence of multiple organs damage, hepatomegaly, ascites, peritoneal seeding, the absence of mature mast cells and other hematopoietic cells in the BM, and elevated serum Tryptase level.

## Introduction

Mastocytosis is defined as infiltration of atypical mast cells (MCs) in one or more organs (1, 2). In the patients with systemic mastocytosis (SM), neoplastic MCs constitute focal or diffuse infiltrates in various internal organs such as bone marrow (BM), spleen, liver and gastrointestinal tract (1). Parwaresch et al., (3) and Horny HP et al., (4) also stated that MC neoplasia is generally confined to the dermis. Cutaneous mastocytoses are called benign mastocytoma when localized and urticaria pigmentosa when disseminated. The generalized mastocytosis involves extra-cutaneous tissue irrespective of skin involvement. 

According to the 2016 updated WHO classification, two major subtypes are Cutaneous Mastocytosis (CM), and Systemic Mastocytosis (SM). In systemic type, neoplastic mast cells involve internal organs with or without cutaneous involvement. SM is subdivided into Indolent SM (ISM), Smoldering SM (SSM), SM with an associated hematologic neoplasm (SM-AHN), aggressive SM (ASM), mast cell leukemia (MCL), mast cell sarcoma, and extra cutaneous mastocytoma (1). Arber DA et al., (5) found that the 2016 edition represented a revision of the prior classification rather than an entirely new classification. Thus, they attempted to incorporate new clinical, prognostic, morphologic, immune-phenotypic, and genetic data that have emerged since the last edition.

WHO recommendations for the diagnosis of SM are considering detection of the following major criteria with at least one major criterion or three minor criteria (1, 6, 7).

Major criterion is characterized by the observation of aggregates (more than 15 cells) of mast cells in bone marrow or extra cutaneous organs. Minor criteria include more than 25% immature spindle-shaped or atypical mast cells in BM or extra cutaneous organs, the presence of KIT D816V point mutation, and phenotypic expression of CD2 or CD25, more than 20 ng/ml serum Tryptase level in the absence of clonal myeloid disorder (1, 6). 

MCL, less than 0.5% of mastocytosis, is the leukemic spreading of atypical mast cells, involving more than 20% of bone marrow hematopoietic cells in aspirate or biopsy, with aleukemic variant that shows less than 10 percent mast cells in WBC count of blood (2). Valentini CG et al., (8) and Swerdlow H et al., (9) described the same viewpoints in the previous parallel studies.

Recently, Valent P et al., (10) classified MCL into two categories in a new study to refine diagnostic criteria and classification of mast cell leukemia (MCL) and myelomastocytic leukemia (MML).They proposed a chronic form without obvious organ damage (no C findings present) and a more aggressive variant, and acute MCL where organ damage (C findings) is present.

Literatures state that c-KIT mutation is the most frequent abnormality encountered and a hallmark of the disease. Mast/stem cell growth factor receptor (SCFR), also known as proto-oncogene c-Kit or tyrosine-protein kinase Kit or CD117, is a receptor tyrosine kinase protein that is encoded by the KIT gene in humans (11). Positive IHC staining for CD117/kit in bone marrow is supposed to be essential for the diagnosis of MCL. We describe one case of MCL with insignificant reactivity for CD117. Joris M et al., (12) and Georgin-Lavialle S (13) also showed that non-KIT D816V mutations are unexpectedly frequent and therefore, complete gene sequencing is necessary. 

The study illustrates a very rare case of aleukemic variant of acute mast cell leukemia with weight loss, ascites and multiple organs involvement including liver, peritoneum and lymph nodes.

## Case Report

A 57-year-old man was referred to our center with the history of anemia and ascites for two years. He complained from weakness, periodic headache, occasional vomiting and significant weight loss. 

His vital signs were within normal ranges on systemic physical examination. Cervical lymphadenopathy was remarkable. Chest and lungs auscultation revealed faded respiratory sounds over middle and lower lobes of the right lung. The liver edge was palpable 3 cm below the right costal margin and evidences of spleen enlargement with mild abdominal distention were identified.

Further evaluations including laboratory tests on serum and ascites fluid, and radiologic investigations were done. Abdominal ultrasound showed multiple para aortic enlarged lymph nodes, splenomegaly (166 mm), and free intraperitoneal fluid. In chest and abdominopelvic CT scan the same findings were distinguished such as hepatosplenomegaly and para aortic lymphadenopathy. Anemia was confirmed by CBC (WBC:6600, Hb:9.8, Plt:307000). Ascites fluid and other laboratory data were unremarkable.

Investigation

During hospitalization, bone marrow aspiration and biopsy were done with simultaneous PBS preparation, and stained by Giemsa stain.

Peripheral blood showed hypochromia, anisocytosis, and poikilocytosis of red blood cells. WBC differential count was within normal range (51% PMN, 32% lymph, 2% monocyte, 1% eosinophil and 14% activated lymphoid cells).

**Figure 1:**
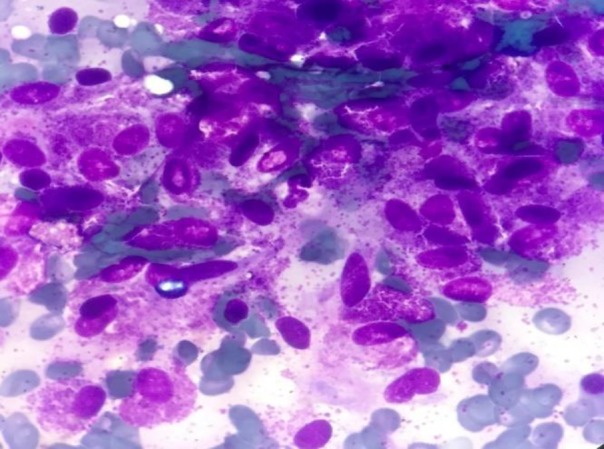
Bone marrow smear showed numerous metachromatic blast cells with vacuolated cytoplasm containing varying numbers of metachromatic granules and spindle-shaped nuclei. (Giemsa, 1000×)

Giemsa-stained bone marrow aspiration revealed many three-dimentional clusters of de-granulated or hypogranulated mast cells with spindle-shaped nuclei, clear or eosinophilic granular cytoplasm, with no other hematopoietic cells presence and mature mast cells absence, supported by Toluidine blue stain (Figure 1).

**Figure 2:**
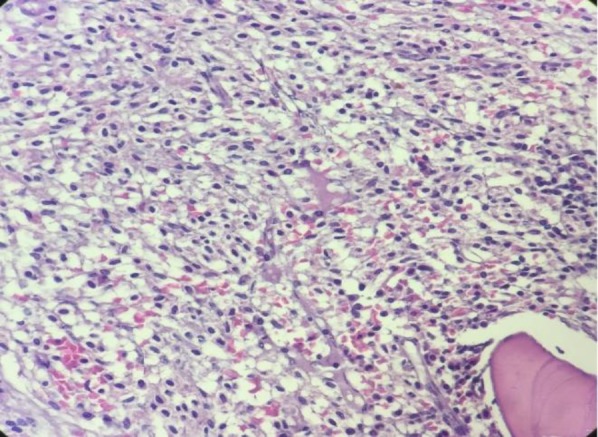
Bone marrow biopsy showed the sheet of spindle-shaped mast cells. (Hematoxylin and eosin, 400×)

**Figure 3:**
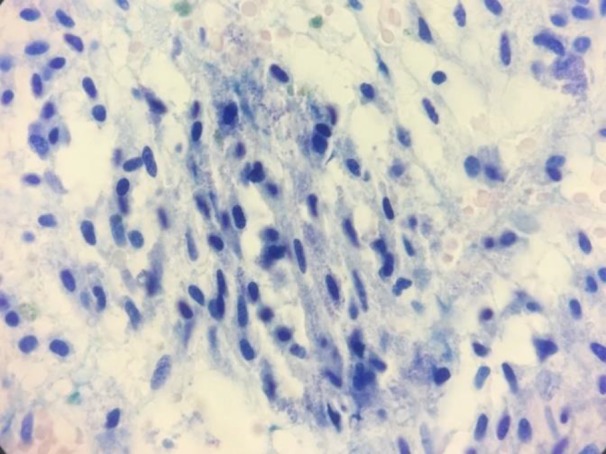
Bone marrow biopsy revealed numerous mast cells with metachromatic granules. (Toluidine blue stain, 400×)

Bone marrow biopsies were stained by H&E, Giemsa, and Toluidine blue stains and showed about 100% cellularity with the aggregates of atypical spindle-shaped mast cells that represent hypogranulation. Several megakaryocytes with dysplastic figures were also identified. Immunohistochemistery evaluation of bone marrow biopsy demonstrated low expression of CD117 and CD45 (Figure 2, 3).

After achieving these findings, the serum Tryptase level was checked in the patient. It was markedly elevated above the normal limit (465 ng/ml).

**Figure 4:**
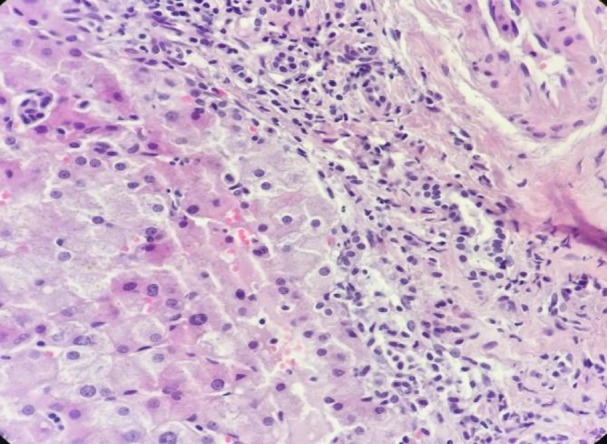
Liver biopsy showed peri-portal mast cell infiltration. (H&E, 400×)

**Figure 5:**
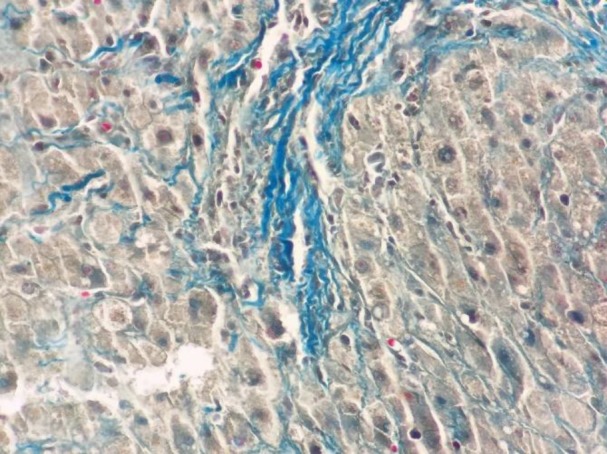
Liver biopsy showed diffuse fibrosis and few metachromatic mast cells. (Trichrome, 400×)

**Figure 6:**
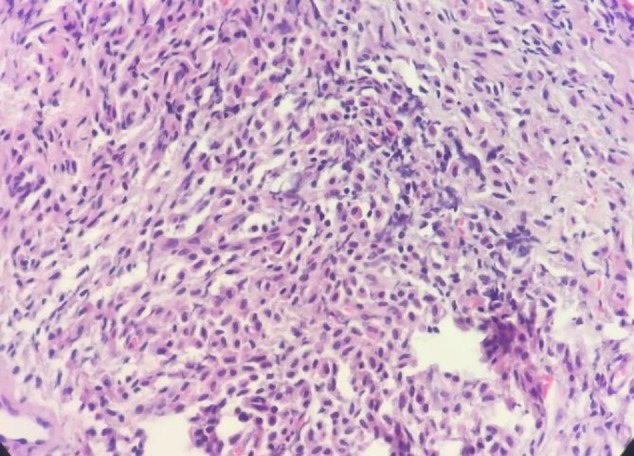
Peritoneal biopsy showed mast cell distribution. (H&E, 100×)

**Figure 7:**
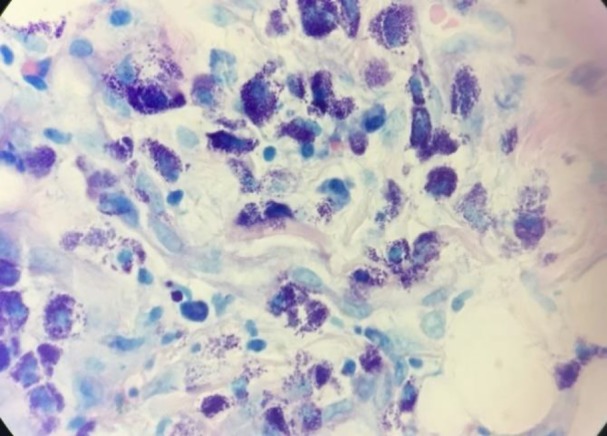
Peritoneal biopsy showed pleomorphic mast cells with metachromatic granules. (Giemsa, 400×)

Due to massive hepatosplenomegay and generalized lymphadenopathy, liver biopsy and lymphadenectomy were done by the surgeon. The specimens were stained by H&E and Giemsa stains. Liver tissue showed dense portal, sinusoidal, and micronodular mast cells infiltration with portal fibrosis. (Figure 4,5). Lymph nodes also showed mast cells distribution through subcapsular and sinus spaces. (Figure 6,7).

Other findings during surgical exploration included spleen enlargement with whitish surface, and peritoneal tumor implants, which was confirmed by biopsy and microscopic pathologic evaluation.

Few days after splenectomy, chemotherapy in combination with cytosine arabinoside and mitoxantrone was used. Multi-organ failure including bone marrow failure developed over few months and the patient died.

The images and information from individual participants are obtained with their informed consent prior to their inclusion in the study. For observing the ethics of this research, the patient anonymity was preserved.

## Discussion

Several MCL is a very rare form of the aggressive systemic mastocytosis, including 0.5% of all mastocytosis (1, 2). The 2016 updated WHO classiﬁcation defined 7 variants: cutaneous mastocytosis, indolent systemic mastocytosis (ISM), systemic mastocytosis (SM) with an associated clonal hematologic non–MC-lineage disease (SMAHNMD), aggressive SM (ASM), MC leukemia (MCL), MC sarcoma, and extracutaneous mastocytoma (1). 

It is characterized by the leukemic proliferation of atypical mast cells, with the frequent multiple organs involvement such as the liver, peritoneum, spleen, and bone marrow (2).  

According to the Jawhar M, et al., study, MCL showed more clinical and biological features of the advanced systemic mastocytosis than acute myeloid leukemia (AML), occurring de novo or secondary to the evolved other advanced SM (14).

Our case was presented with abdominal organomegally, ascites, weight loss and anemia without past history of mastocytosis. Few previous studies reported systemic mastocytosis with the combination of these clinical signs. Zhang XY et al., (15) reported an unusual case of aggressive systemic mastocytosis mimicking hepatic cirrhosis with the main clinical features of hepatomegaly, portal hypertension, ascites, and fibrosis. Narayanan MN et al., (16) reported a case of systemic mastocytosis (SM) presenting as ascites and portal hypertension, that the disease progressed rapidly and recurrent massive ascites was a dominant problem. Jawhar M et al., (14) in a case series study showed that 8 of 16 cases (50%) had marked splenomegaly (≥1200 ml).

MCL diagnosis requires the presence of SM criteria with additional features including involvement of bone marrow (BM) and/or blood by at least 20% atypical mast cells as well as the neoplastic mast cell infiltration of extra-cutaneous organs (2). The SM diagnosis is established if at least a major criterion (aggregates of mast cells in bone marrow or extra cutaneous organs) or at least 3 minor criteria (more than 25% immature spindle-shaped or atypical mast cells in BM or extra cutaneous organs, the presence of KIT D816V point mutation, phenotypic expression of CD2 or CD25, and more than 20 ng/ml serum Tryptase level in the absence of clonal myeloid disorder) are identified (1).

Our case fulfilled the diagnostic criteria, as involvement of bone marrow by multifocal dense infiltrates of mast cells with spindle-shaped and atypical morphology. Marked elevated serum Tryptase level exceeding 20 ng/ml also supported diagnosis as a minor criterion.  Suh MC et al., (17) stated that the presence of abnormal mast cells (premature promastocytes) with marked morphologic atypia, as the predominant cell type is seen in the aggressive type of systemic mastocytosis and related to the poor prognosis of the disease.

A review article by Giorgin-Lavialle S, et al., suggests that threshold >20% mast cell of bone marrow should be confirmed by the qualitative and quantitative cytological analysis of bone marrow aspirate (2).

We verified the diffuse infiltration of atypical mast cells without mature mast cells in bone marrow aspirate. The biopsy of our patient was confirmed by the positive Giemsa and Toluidine blue stains of metachromatic granules through leukemic cells. 

Typically, peripheral mast cells exceed 10% of all blood nucleated cells; however, laeukemic variants exist and its diagnosis is made if the number of circulating MCs is less than 10% [18]. Our case revealed no mast cell on PBS. Thus, the diagnosis of aleukemic variant of MCL was considered for the patient. Suh MC et al., (17) represented a highly aggressive de novo aleukemic variant of mast cell leukemia without KIT D816V mutation. 

Mast cell leukemia is very aggressive. The poor prognosis with a median survival of 2 months has been shown in some studies (19). Recently, a discrepancy between chronic and acute MCL has been proposed according to the involvement of non-MC lineages, and aggressiveness of the disease (C findings). The chronic MCL is defined by the absence of C findings, whereas in acute (classical) MCL, at least one C finding is detected (i.e. indicative of organ damage). Only a few patients with acute MCL survived more than1 year even after chemotherapy or stem cell transplantation (1, 2). 

The C findings include cytopenia, hepatomegaly with ascites and impaired liver function, palpable splenomegaly, malabsorption with hypoalbuminemia and weight loss, skeletal lesions, and life-threatening organ damage in other organ systems that is caused by the local MC infiltration in tissues (1). 

Clinical, radiological and laboratory investigations in our reported case showed the presence of multiple C findings and organ damages, suggesting the diagnosis of acute form of MCL and recommending the progressive disease course.

According to all clinicopathological findings of the patient, we confronted a very rare case of aleukemic acute mast cell leukemia with multiple organ damage, ascites, and the expression of CD117/KIT mutation. These manifestations of disease showed its aggressive behavior. Despite the surgical treatment and chemotherapy patient died a few months later.

## Conclusion

We presented a case of aleukemic variant of acute MCL with highly aggressive disease progression. We suggested possible risk factors for the prognosis like the presence of multiple organ damages, symptoms of aggressive disease, marked hepatomegaly, ascites, peritoneal seeding, the absence of mature mast cells and other hematopoietic cells in the BM, elevated serum Tryptase level, and the expression of CD117/KIT mutation. In the absence of typical skin lesions, systemic mastocytosis diagnosis can be challenging. Early diagnosis may give the patient a chance for the early treatment and more survival time. Acquaint with the characteristic morphology and classification of MCL based on the novel criteria is critical for guiding diagnosis in the right direction.
